# Identification of Key Pathways and Genes Related to Immunotherapy Resistance of LUAD Based on WGCNA Analysis

**DOI:** 10.3389/fonc.2021.814014

**Published:** 2022-01-06

**Authors:** Weina Yu, Fengsen Liu, Qingyang Lei, Peng Wu, Li Yang, Yi Zhang

**Affiliations:** ^1^ Biotherapy Center and Cancer Center, The First Affiliated Hospital of Zhengzhou University, Zhengzhou, China; ^2^ Henan Key Laboratory for Tumor Immunology and Biotherapy, Zhengzhou University, Zhengzhou, China; ^3^ State Key Laboratory of Esophageal Cancer Prevention and Treatment, Zhengzhou University, Zhengzhou, China; ^4^ School of Life Sciences, Zhengzhou University, Zhengzhou, China

**Keywords:** LUAD, WGCNA, TIDE, immunotherapy resistance, cancer stem cell

## Abstract

Immunotherapy resistance is a major barrier in the application of immune checkpoint inhibitors (ICI) in lung adenocarcinoma (LUAD) patients. Although recent studies have found several mechanisms and potential genes responsible for immunotherapy resistance, ways to solve this problem are still lacking. Tumor immune dysfunction and exclusion (TIDE) algorithm is a newly developed method to calculate potential regulators and indicators of ICI resistance. In this article, we combined TIDE and weighted gene co-expression network analysis (WGCNA) to screen potential modules and hub genes that are highly associated with immunotherapy resistance using the Cancer Genome Atlas (TCGA) dataset of LUAD patients. We identified 45 gene co-expression modules, and the pink module was most correlated with TIDE score and other immunosuppressive features. After considering the potential factors in immunotherapy resistance, we found that the pink module was also highly related to cancer stemness. Further analysis showed enriched immunosuppressive cells in the extracellular matrix (ECM), immunotherapy resistance indicators, and common cancer-related signaling pathways in the pink module. Seven hub genes in the pink module were shown to be significantly upregulated in tumor tissues compared with normal lung tissue, and were related to poor survival of LUAD patients. Among them, THY1 was the gene most associated with TIDE score, a gene highly related to suppressive immune states, and was shown to be strongly expressed in late-stage patients. Immunohistochemistry (IHC) results demonstrated that THY1 level was higher in the progressive disease (PD) group of LUAD patients receiving a PD-1 monoclonal antibody (mAb) and positively correlated with SOX9. Collectively, we identified that THY1 could be a critical biomarker in predicting ICI efficiency and a potential target for avoiding tumor immunotherapy resistance.

## Introduction

Chemotherapy, radiotherapy, and surgery have dominated the cancer treatment field for years and did acquire considerable effects. However, these traditional therapies have noticeable limitations in treating patients with late-stage or metastatic malignant tumors and often cause severe side effects. To solve current problems, immunotherapies are emerging as promising methods by rebuilding immunosurveillance and stimulating tumor immune elimination (1). T cells are primary participants in tumor-associated immune response. Their infiltration, ratio, and functions influence antitumor immune response (2). Immune checkpoints [cytotoxic T lymphocyte-associated antigen-4 (CTLA-4), programmed cell death protein 1 (PD-1)] and immune checkpoint-related ligands [immune checkpoint-related ligands such as programmed cell death ligand 1 (PD-L1)] are critical parts in this process that promote or inhibit T-cell activity (3). As a result, ICIs are considered a promising strategy in reactivating antitumor immune responses by blocking immune checkpoints/immune checkpoint ligands. In recent decades, ICI therapy has been shown to be effective in patients with refractory tumors, especially for those late-stage patients who were unresponsive to traditional treatments (4). The United States Food and Drug Administration approved its application in solid tumors in 2011 (5). Anti-PD-1/PD-L1 therapy increased the overall survival to 40–50% at 5 years in advanced melanoma patients and is also becoming a promising method for non-small-cell lung cancer (NSCLC) patients (6, 7). The KEYNOTE‐042 study indicated that in NSCLC patients whose PD-L1 tumor proportion score was ≥1%, pembrolizumab monotherapy prolonged their survival for longer than with traditional chemotherapy (8). In addition, dual blockage of PD-1 and CTLA-4 using nivolumab and ipilimumab also showed better effects compared with chemotherapy (9).

However, ICI did not show the same effects in all malignant tumor patients, and its response rate in an unselected population as single agent is below 20% (7, 10). More than one-half of patients are either unresponsive, or they relapse after a period of response, which seriously limits the effectiveness of ICI (11). ICI resistance can be generally divided into two sorts: primary resistance in which patients experience no significant effect in the initial period of ICI treatment; and secondary resistance (acquired drug resistance) in which initially, ICI can slow tumor progression, but patients later develop treatment failure (12). Two primary reasons for ICI resistance are the dysfunction of tumor infiltrating cytotoxic T cells and immunosuppressive factors, which hinder T cells from entering the tumor microenvironment (TME) (13, 14). Although many mechanisms were reported to be accountable for ICI resistance, there is still not an efficient method to predict it. Therefore, identification of predictive biomarkers for ICI response is crucial for determining appropriate patients for and predicting efficacy of ICI. TIDE is a computational method developed by Jiang et al. and is used for forming a tumor immune evasion model and calculating potential regulators and indicators in ICI resistance through integrating the expression features of T cell dysfunction and T cell exclusion, as well as pre-treatment tumor profiles (15). To identify potential biomarkers associated with immunotherapy, especially ICI resistance, WGCNA was also adopted in this study. WGCNA is a newly developed bioinformatics method to process medical data. Various researchers have utilized this method to analyze the gene expressions in different samples, depict correlations, and identify potential co-expressed genes or targets (16–18).

Self-renewal capacity and long-term persistence are critical properties of cancer stem cells (CSCs), and help them to maintain immune evasion, epithelial-mesenchymal transition (EMT), signaling pathway regulation, multidrug resistance, and pro-tumor immunity (19). CSCs express low levels of cancer-associated antigens, immune stimulatory molecules (CD86, CD40, and MHC II), and high levels of immune checkpoint proteins (20). Recent studies put forward that cancer cell stemness could be represented by clusters of core genes (21–23). Based on these gene sets, Miranda et al. found pervasive negative associations between cancer cell stemness and anticancer immunity (24). They showed that stemness led to higher intratumoral heterogeneity and restrained antitumor immune response, which result in poor outcome for malignant tumor patients. Research showed that complex interactions between CSCs and immune cells in the TME-induced immunotherapy resistance by promoting anti-apoptotic pathways, inducing EMT, and enhancing immune tolerance (25–27).

After taking into consideration both TIDE score and stemness index, we identified a potential gene module (pink) which was highly related to T cell dysfunction and cancer cell stemness. Further analysis revealed strong enrichment of ECM remodeling, EMT, tumor invasion, tumor growth, and a positive relationship to immunosuppressive markers in the pink module. According to the gene significance of TIDE (GS.TIDE), we selected nine genes and observed their higher levels in tumor tissues and LUAD patients with poorer survival. Among them, THY1 (Thy-1 cell surface antigen) was selected as the key gene and was shown to be related to tumor stage, survival, and immune states of LUAD patients. Analysis of clinical samples showed a positive correlation between THY1 and classical cancer stemness marker SOX9. Moreover, the highest levels of THY1 were found in the PD group of PD-1 mAb-treated LUAD patients. These results provide the fundamental basis for further research of immunotherapy resistance and the discovery of new therapeutic target to refine ICI treatments of LUAD patients.

## Materials and Methods

### Data Processing

We obtained gene expression data (FPKM format) and relevant clinical characteristics of 526 LUAD samples from the Cancer Genome Atlas (TCGA) database (https://www.cancer.gov/tcga). After excluding patients that lacked key clinical information, 513 patients were retained. TPM format conversion was performed for gene expression data in FPKM format for further analysis. We also obtained data for gene expression and survival of LUAD patients (GSE72094) from Gene Expression Omnibus (https://www.ncbi.nlm.nih.gov/geo/). A total of 398 samples with complete clinical information were utilized in further analysis.

### Immunotherapy Response Prediction and Stemness Index

TIDE score was shown to be more effective and accurate than current methods in predicting the immunotherapy response of melanoma and lung cancer patients. We downloaded LUAD patient-related TIDE scores from (http://tide.dfci.harvard.edu) (15). To bring cancer stemness into analysis, we used single-sample gene set enrichment analysis (ssGSEA) to process stemness-related gene sets (Benporath_ES1, Benporath_ES2, Bhattacharya_hESC, Shats_iPSC, Shats_Consensus, Benporath_Sox2, Kim_Myc, Smith_Human.Epithelial_ASC, Palmer_2012, Proliferative_Ben_Porath, Curated_genes) in malignant tumors obtained from previous articles (21–23, 28–32). The above data were included in the WGCNA analysis according to a previous report (33).

### Constructing Weighted Gene Co-Expression Networks and Identifying Modules Associated With Immunotherapy Resistance Score and Stemness Score of LUAD Patients

WGCNA is used to group correlated genes pairwise into a model or network according to their similar expression profiles (34). In this article, WGCNA was adopted to identify the modules correlated with immunotherapy resistance and cancer stemness. Further filtration of 513 LUAD patients found that six patients lacked TIDE values or were outliers according to the original clustering tree at a height of 150,000. As a result, we ultimately included 507 samples in the WGCNA analysis. We chose the soft threshold β = 4 (scale free R^2^ = 0.88) to construct a co-expression network. We then transformed the adjacency matrix into a topological overlap matrix to quantitatively describe the similarity. Next, we used cutreeDynamic function to perform the gene hierarchical clustering dendrograms and identify various co-expression modules. Correlations between modules and TIDE scores or other characteristics were assessed by Spearman test.

### Identification of Hub Genes

GS and module membership (MM) were adopted to filter hub genes in the pink module. GS stands for the level of correlation between gene expression and designated features. MM stands for the correlation of the module eigengene and the gene expression profile. GS.TIDE represents the relevance of each module to the TIDE score. Among all the modules, the pink module had the highest GS.TIDE, which means the highest correlation with immune evasion. Therefore, we selected top 20 genes according to GS.TIDE for protein-protein interaction (PPI) network analysis and nine genes for further analysis. Among them, THY1 was the gene most associated with TIDE score in the pink module.

### Protein-Protein Interaction Analysis of Top 20 Hub Genes

We performed a PPI analysis with top 20 hub genes using GeneMANIA website (https://genemania.org/search/homo-sapiens/) to depict their relationship in Co-expression, Physical Interactions, Co-localization, and Pathways.

### Gene Ontology and Kyoto Encyclopedia of Genes and Genomes Enrichment Analysis

Clusterprofiler R package was adopted in Gene Ontology (GO) and Kyoto Encyclopedia of Genes and Genomes (KEGG) enrichment analysis using genes in the pink module. We used “adjusted *p* < 0.05” to identify significant GO terms and KEGG terms.

### Cell Infiltration Analysis

xCell (http://xcell.ucsf.edu/) was used to obtain the relative expression level of 64 types of cells in the tumor microenvironment of LUAD patients using mRANseq data (TPM format) from TGCA in R software.

### Pathway Enrichment of the Pink Module

Four gene set lists were downloaded from the nanoString website (https://www.nanostring.com/): the nCounter PanCancer Pathways panel, the nCounter^®^ PanCancer Immune Profiling panel, the nCounter PanCancer Progression panel, and the nCounter^®^ PanCancer IO 360™ Panel. These panels include detailed listings of genes, corresponding pathways, and immune types. ssGSEA and heatmap packages were used to analyze these four panels and displayed the results by LUAD patients.

### Kaplan-Meier Survival Analysis

Gene expression data and corresponding clinical information from GSE72094 dataset were used to analyze the survival rate of each gene. We utilized Kaplan-Meier curve and log-rank test to estimate the prognostic significance of nine hub genes. We divided each gene into two groups according to the best cut point method using “surv_cutpoint” algorithm of the survminer R package.

### Immune Subtypes and Clinical Characteristics Analysis

According to the research of Thorsson et al., the intratumor immune states could be divided into six subtypes: C1 (wound healing), C2 (IFN-γ dominant), C3 (inflammatory), C4 (lymphocyte depleted), C5 (immunologically quiet), and C6 (TGF-β dominant) (35). C1 has higher expression of angiogenic genes and Th2-type adaptive immune infiltration. C2 shows high M1/M2 macrophage polarization and activated function of antitumor T cells. C3 is related to Th17 and Th1 cells and could suppress tumor cell proliferation. C4 displays a prominent M2 macrophage signature and suppressed Th1. C5 exhibits the lowest lymphocyte and highest M2 responses. The highest TGF-β signature is the feature of C6. We evaluated the expression level of THY1 on these six subtypes. We also assessed THY1 expression in different clinical stages of LUAD patients using gene expression data and clinical information from the TCGA dataset. Wilcoxon test was adopted to compare differences between groups.

### Clinical Samples

Human LUAD tumor tissues were acquired from the First Affiliated Hospital of Zhengzhou University in 2020. All patients were pathologically diagnosed with LUAD through surgery or percutaneous pulmonary biopsy and failed chemotherapy or targeted therapy. All patients had a lung computerized tomography (CT) scan before receiving at least three cycles of PD-1 mAb treatment (baseline). Lung CT scan was also used to evaluate the therapeutic effects after using PD-1 mAb (first/second). The clinical information of the patients is shown in **Table 1**.

**Table 1:** The summary clinical information of the samples.

Characteristics	LUAD Patients (n = 6)
Age, year *(mean ± SD)*	63.50 ± 8.12
Male, *n (%)*	2 (*33.3*)
Female, *n (%)*	4 (*66.7*)
TNM stage, *n (%)*	
I	0 (*0*)
II	1 (*16.7*)
III	3 (*50*)
IV	2 (*33.3*)
Outcome, *n*	
PD	2
SD	2
PR	2

SD, standard deviation; LUAD, lung adenocarcinoma; PD, progressive disease; SD, stable disease; PR, partial response.

### Immunohistochemistry

All patients agreed to informed consent before specimen collection. Tissues were fixed with 4% paraformaldehyde and embedded in paraffin. IHC was performed according to previous research (36). Tumor tissue slides were incubated with anti-human THY1 (ABCAM, Rabbit mAb, CAT: ab133350, 1:50) and anti-human SOX9 (ABCAM, Rabbit mAb, CAT: ab185966, 1:1,000) at 4°C for overnight. Then, the slides were incubated with horseradish peroxidase-conjugated anti-rabbit/mouse antibody (ZSGB-BIO, SP-9000) for 30 min at room temperature. Subsequently, diaminobenzidine (ZSGB-BIO, ZLI-9018) and hematoxylin were used to visualize the protein and cell nucleus. Vectra Polaris Multispectral Imaging and Whole Slide Scanning System (PerkinElmer, Vectra) was used to screen the slides. IHC staining score of each protein was evaluated by imageJ software using IHC toolbox.

### Statistical Analysis

Wilcoxon test is used to compare the differences between groups of classified variable. Spearman analysis is used to calculate the correlation between continuous variables. The above collected data was analyzed using R software version 3.6.1 (https://www.r-project.org). Differences between groups were analyzed using t test. Pearson’s correlation analysis was used to calculate correlation coefficients (r) using Graphpad Prism 7. With all statistical methods, *p* < 0.05 was considered statistically significant.

## Result

### WCGNA Analysis and Modules Significance Calculation

**Figure 1:**
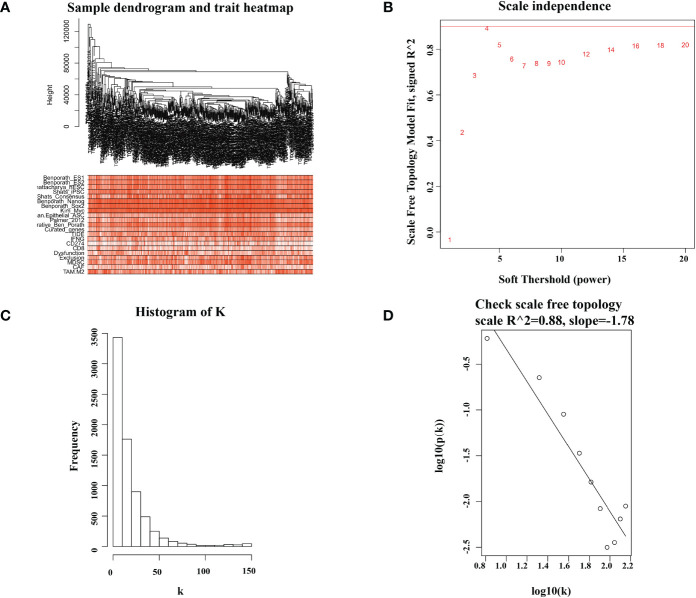
Sample dendrogram and soft-thresholding values estimation. **(A)** Sample dendrogram and trait heatmap of 507 samples. The upper 12 traits are representative marker of cancer stemness, and the lower 9 traits are common evaluation index of immunotherapy. **(B)** Analysis of the scale-free index for various soft-threshold powers (β). **(C, D)** The scale free topology when β = 4.

**Figure 2:**
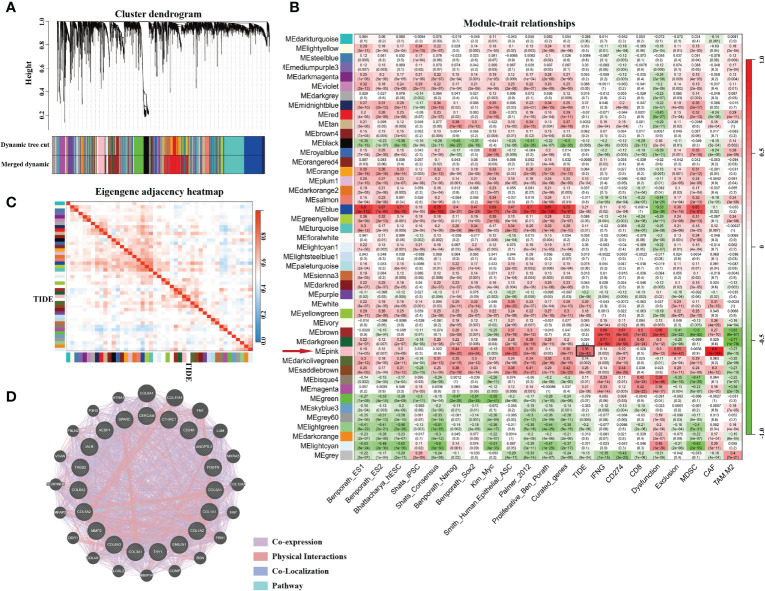
The genes enrichment and module identification. **(A)** Hierarchical clustering dendrogram of co-expressed genes in LUAD patients. A total of 45 modules were identified, and each colored row stands for a module with a cluster of highly connected genes. **(B)** Correlation coefficient between consensus module eigengenes and TIDE score, cancer stemness, or other immune-related characteristics. **(C)** Heatmap plot of the adjacent modules. **(D)** Protein–protein interactions of top 20 hub genes. The strength of the relationship is associated with the thickness of the colorful lines.

The gene expression matrix of 7,298 preprocessed genes derived from LUAD patients in the TCGA dataset was used for WGCNA with R package. After excluding patients that lacked critical characteristics, 507 samples were utilized for further analysis. Sample dendrogram and trait heatmap are displayed in **Figure 1A**. To ensure high-scale independence (near 0.9), we set β = 4 in this analysis (**Figure 1B**). After obtaining the β value, we constructed an adjacency matrix and topological overlap matrix (**Figures 1C, D**). On the basis of average hierarchical clustering and dynamic tree clipping, we obtained 45 modules in total (**Figure 2A**). Then, we calculated the correlation coefficients between each module and the sample characteristics related to TIDE, cancer stemness markers, and various immune-related characteristics (**Figures 2B, C**). According to GS.TIDE, we found that the pink module had the highest correlation with TIDE (cor = 0.56; *p* = 2e-43). In addition, the pink module showed a strong correlation with various cancer stemness markers and immunosuppressive features, such as Exclusion (cor = 0.55; *p* = 4e-42) and cancer-associated fibroblast (CAF) (cor = 0.9; *p* = 3e-189) (**Figure 2B**). As a result, this module was selected as a critical module for the possible relationship between cancer stemness and immunotherapy resistance. GS.TIDE was used to filter the top 20 hub genes in the pink module (**Table 2**). We adopted the PPI network with top 20 hub genes using GeneMANIA and found a strong relationship in expression, physical interaction, and pathways between hub genes (**Figure 2D**).

**Table 2:** The top 20 hub genes in pink module.

Gene symbol	Module color	GS.TIDE	p.GS.TIDE		MM.PINK	p.MM.pink
THY1	pink	0.565112	4.07E-44		0.863729	2.10E-152
COL5A1	pink	0.561903	1.56E-43		0.909754	6.08E-195
COL1A2	pink	0.545879	1.03E-40		0.900204	1.86E-184
COL3A1	pink	0.53608	4.63E-39		0.871879	1.08E-158
COL1A1	pink	0.536067	4.65E-39		0.85819	2.33E-148
COL5A2	pink	0.535168	6.55E-39		0.894479	1.14E-178
MMP2	pink	0.528323	8.60E-38		0.870475	1.40E-157
COL6A3	pink	0.52624	1.86E-37		0.880475	8.24E-166
EMILIN1	pink	0.520856	1.34E-36		0.828496	2.91E-129
CTHRC1	pink	0.519758	1.99E-36		0.714135	2.97E-80
CERCAM	pink	0.519243	2.40E-36		0.686384	7.24E-72
SPARC	pink	0.517456	4.56E-36		0.898107	2.69E-182
POSTN	pink	0.516439	6.56E-36		0.803405	8.62E-116
ANGPTL2	pink	0.51307	2.17E-35		0.835944	1.09E-133
COL6A2	pink	0.511227	4.16E-35		0.794625	1.57E-111
AEBP1	pink	0.508668	1.02E-34		0.884474	2.60E-169
THBS2	pink	0.506547	2.13E-34		0.877036	6.73E-163
ISLR	pink	0.504474	4.35E-34		0.794421	1.96E-111
CD248	pink	0.499077	2.74E-33		0.719266	6.47E-82
BGN	pink	0.498091	3.81E-33		0.854806	5.69E-146

GS, gene significance; MM, module membership.

### Functional Annotation of Genes in the Pink Module

**Figure 3:**
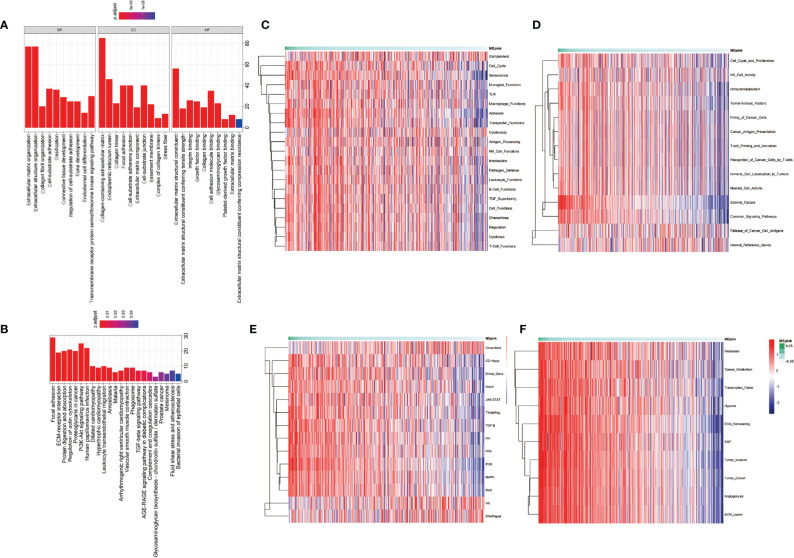
Enrichment analysis of the significant genes in the pink module. **(A)** Gene ontology enrichment including biological process (BP), cellular component (CC), and molecular function (MF). **(B)** KEGG enrichment of significant genes in pink module. **(C–F)** Pathway enrichment of significant genes in pink module. **(C)** is the pathways that participate in immune profiling. **(D)** is the pathways in IO.unity. **(E)** is the common pathways in human physiological process. **(F)** is the pathways of cancer progression.

The pink module contains 274 genes. The results of the GO enrichment analysis and KEGG enrichment analysis of these 274 genes are shown in **Figures 3A, B**. Results showed that genes in the pink module were strongly enriched in the ECM organization. Enriched items in the cellular component were also related to the ingredients of ECM and focal adhesion (**Figure 3A**). Results from KEGG analysis showed a similar conclusion (**Figure 3B**). Top 15 GO items of the hub genes in the pink module are shown in **Table 3**.

**Table 3:** The top 15 GO items of genes in the pink module.

GO-ID	*p*-value	Description	Genes in the test list
5578	3.30E-20	Proteinaceous extracellular matrix	*POSTN|SPARC|LUM|MMP2|BGN|COL1A1|ADAMTS2|COL3A1|VCAN|COL1A2|COL5A1|COL5A2|TIMP2|COL6A3|ANGPTL2|EMILIN1|FBN1*
31012	7.25E-20	Extracellular matrix	*POSTN|SPARC|LUM|MMP2|BGN|COL1A1|ADAMTS2|COL3A1|VCAN|COL1A2|COL5A1|COL5A2|TIMP2|COL6A3|ANGPTL2|EMILIN1|FBN1*
44421	3.67E-15	Extracellular region part	*POSTN|SPARC|LUM|MMP2|BGN|AEBP1|INHBA|COL1A1|ADAMTS2|COL3A1|VCAN|COL1A2|COL5A1|COL5A2|TIMP2|COL6A3|ANGPTL2|EMILIN1|FBN1*
44420	2.27E-13	Extracellular matrix part	*COL1A1|COL3A1|SPARC|COL1A2|COL5A1|LUM|COL5A2|TIMP2|COL6A3|FBN1*
5583	3.82E-13	Fibrillar collagen	*COL1A1|COL3A1|COL1A2|COL5A1|LUM|COL5A2*
5201	6.99E-13	Extracellular matrix structural constituent	*COL1A1|COL3A1|COL1A2|COL5A1|LUM|COL5A2|BGN|EMILIN1|FBN1*
30199	1.47E-12	Collagen fibril organization	*COL1A1|ADAMTS2|COL3A1|COL1A2|COL5A1|LUM|COL5A2*
48856	1.86E-12	Anatomical structure development	*PDGFRB|TAGLN|POSTN|SPARC|UNC5B|MMP2|BGN|AEBP1|THY1|INHBA|ANTXR1|COL1A1|ACTA2|ADAMTS2|COL3A1|VCAN|COL1A2|COL5A1|COL5A2|ITGA11|TIMP2|COL6A3|FBN1*
5576	2.27E-12	Extracellular region	*POSTN|SPARC|LUM|MMP2|BGN|AEBP1|INHBA|THBS2|COL1A1|ADAMTS2|COL3A1|VCAN|COL1A2|COL5A1|COL5A2|TIMP2|COL6A3|ANGPTL2|EMILIN1|MXRA5|FBN1*
30198	3.94E-12	Extracellular matrix organization	*COL1A1|ADAMTS2|POSTN|COL3A1|COL1A2|COL5A1|LUM|COL5A2|EMILIN1*
32502	5.15E-12	Developmental process	*PDGFRB|TAGLN|POSTN|SPARC|UNC5B|MMP2|BGN|AEBP1|THY1|INHBA|ANTXR1|COL1A1|ACTA2|ADAMTS2|COL3A1|VCAN|COL1A2|COL5A1|COL5A2|ITGA11|TIMP2|COL6A3|ANGPTL2|FBN1*
5581	5.15E-12	Collagen	*COL1A1|COL3A1|COL1A2|COL5A1|LUM|COL5A2|COL6A3*
7275	1.35E-11	Multicellular organismal development	*PDGFRB|TAGLN|POSTN|SPARC|UNC5B|MMP2|BGN|AEBP1|THY1|INHBA|COL1A1|ACTA2|ADAMTS2|COL3A1|VCAN|COL1A2|COL5A1|COL5A2|ITGA11|TIMP2|COL6A3|ANGPTL2|FBN1*
48731	5.21E-11	System development	*PDGFRB|TAGLN|POSTN|SPARC|UNC5B|MMP2|BGN|AEBP1|THY1|INHBA|COL1A1|ADAMTS2|COL3A1|VCAN|COL1A2|COL5A1|COL5A2|ITGA11|TIMP2|COL6A3|FBN1*
43062	1.49E-10	Extracellular structure organization	*COL1A1|ADAMTS2|POSTN|COL3A1|COL1A2|COL5A1|LUM|COL5A2|EMILIN1*

**Figure 4:**
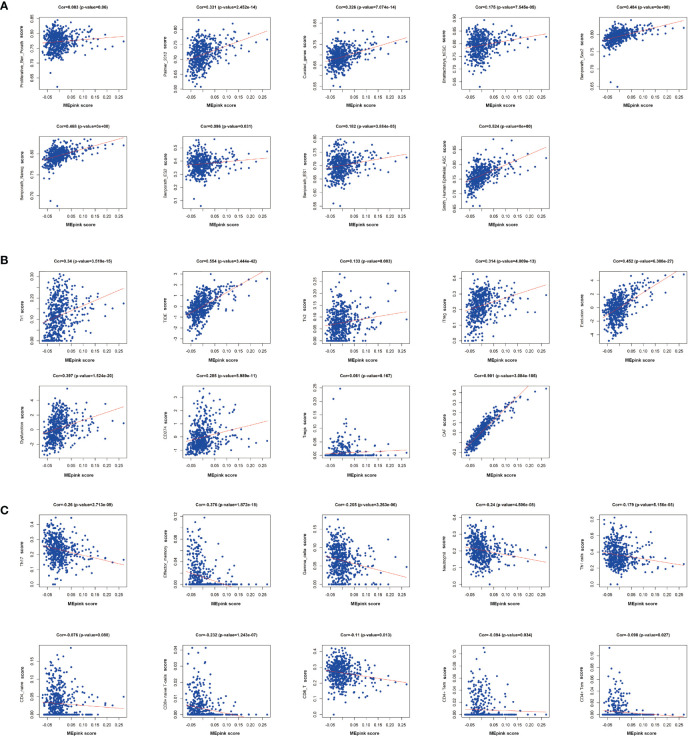
Correlation of significant genes in pink modules with stemness and immunotherapy resistance. **(A)** Correlation analysis of pink modules with markers of cancer stemness. **(B)** Correlation analysis of pink modules with immune suppressive markers. **(C)** Correlation analysis of pink modules with immune stimulative markers. Cor, correlation.

To further investigate the characteristics of these genes, we utilized gene set lists downloaded from nanoString and identified higher enrichment of genes in the immunosuppressive items and tumor-associated pathways and functions (**Figures 3C–F**). For example, the pink module was associated with senescence, adhesion, and transporter functions and was also enriched with tumor intrinsic factors and stromal factors. A cluster of cancer-related pathways were involved, such as JAK-STAT, Wnt, PI3K, MAPK, and RAS. More importantly, the enrichment of metastasis, cancer metabolism, ECM remodeling, EMT, tumor invasion, tumor growth, angiogenesis, and ECM layers was heightened in the pink module. Highly positive correlations were found between the pink module and cancer stemness markers, such as Benporath-Nanog (Cor = 0.468, *p* = 0e+00), Palmer_2012 (Cor = 0.331, *p* = 2.452e-14), and Curated_genes (Cor = 0.326, *p* = 7.074e-14) (**Figure 4A**).

It has been reported that the occurrence of immunotherapy resistance is associated with the upregulation of suppressive characteristics in TME and downregulation of immune stimulators (37). To support the hypothesis that the pink module is associated with immune evasion, we performed a correlation analysis between the pink module and immunosuppressive markers or immunostimulatory makers. We found that pink module genes are positively correlated with immunosuppressive markers and cells, such as Tr1, Th2, iTreg, CAF, and CD274 (**Figure 4B**). Correspondingly, immune-stimulating cells and markers, like Th17, effector-memory and Gamma-delta T cells, and neutrophils, were negatively correlated with the pink module (**Figure 4C**). These results demonstrated that the pink module is a set of genes that potently correlated with cancer stemness and immunosuppressive characteristics.

### Identification and Validation of Hub Genes

**Figure 5:**
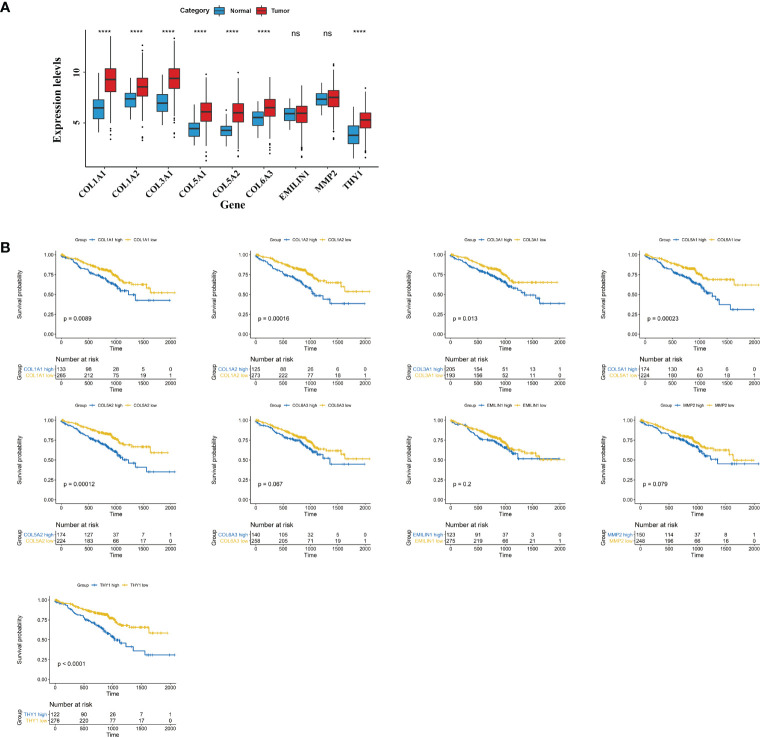
Clinical characteristics of hub genes in pink module. **(A)** mRNA expressions of nine hub genes in cancer tissues of LUAD and normal lung tissues. **(B)** Survival plots of the significant genes by Kaplan Meier test. The data were obtained from the GEO website. ns, no significance; ****p < 0.001.

In *WCGNA Analysis and Modules Significance Calculation* we filtered the top 20 hub genes in the pink module according to GS.TIDE. To shrink the range, we ranked genes on the base of GS.TIDE and selected nine genes from them (*THY1*, *COL5A1*, *COL1A2*, *COL3A1*, *COL1A1*, *COL5A2*, *MMP2*, *COL6A3*, *EMILIN1*). The expression levels and clinical information of these nine genes were obtained from the TCGA and GEO database. Higher expression levels were found in the tumor tissues than in the corresponding normal tissues, except for *EMILIN1* and *MMP2* (**Figure 5A**). We then used a Kaplan-Meier plotter to analyze patient survival and found that *THY1*, *COL5A1*, *COL1A2*, *COL3A1*, *COL1A1*, *COL5A2*, *COL6A3* exhibited excellent diagnostic efficiency in LUAD patients (**Figure 5B**). These results indicated the strong clinical significance of *THY1*, *COL5A1*, *COL1A2*, *COL3A1*, *COL1A1*, *COL5A2*, *COL6A3*.

### Clinical Characteristics of THY1

**Figure 6:**
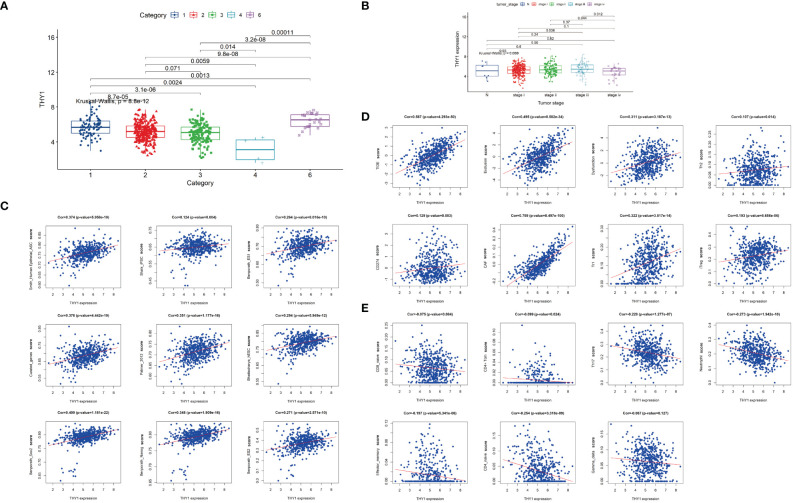
Characteristics of THY1 in LUAD patients. **(A)** THY1 expression of LUAD patients in six immune subtypes: C1, wound healing; C2, IFN-γ dominant; C3, inflammatory; C4, lymphocyte depleted; and C6. TGF-b dominant. **(B)** mRNA expression levels of THY1 in different tumor clinical stages (N and stage i-iv) of LUAD patients. **(C)** Correlation analysis of THY1 and various cancer stemness features. **(D)** Correlation analysis of THY1 with immunosuppressive markers. **(E)** Correlation analysis of THY1 with immunoactivating markers. Cor, correlation.

To find a more targeted gene, we filtered these hub genes according to GS.TIDE, expression levels in cancer, and survival probability, and selected THY1 as the key gene in the pink module. We then analyzed THY1 based on the theory of Thorsson et al. and observed that THY1 was highly expressed in the wound-healing subtype (C1) and TGF-βdominant subtype (C6) (**Figure 6A**), which means that THY1 is an immunosuppressive participant in malignant tumors. Moreover, clinical characteristics analysis showed that tumor stages were highly associated with THY1 expressions, which were highest in LUAD patients in stage iii (**Figure 6B**). Correlation analysis revealed that THY1 had a strong positive correlation with cancer stemness markers, as well as immunosuppressive markers and cells, especially with the TIDE score (Cor = 0.578, *p* = 4.293e-50) (**Figures 6C, D**). The negative correlation between THY1 and immune stimulators were also apparent (**Figure 6E**). These results implied that THY1 mediates immunotherapy resistance by enhancing immunosuppressive function as well as limiting the antitumor effects of immune stimulators.

### Clinical Validation of THY1 and SOX9 in LUAD Patients

**Figure 7:**
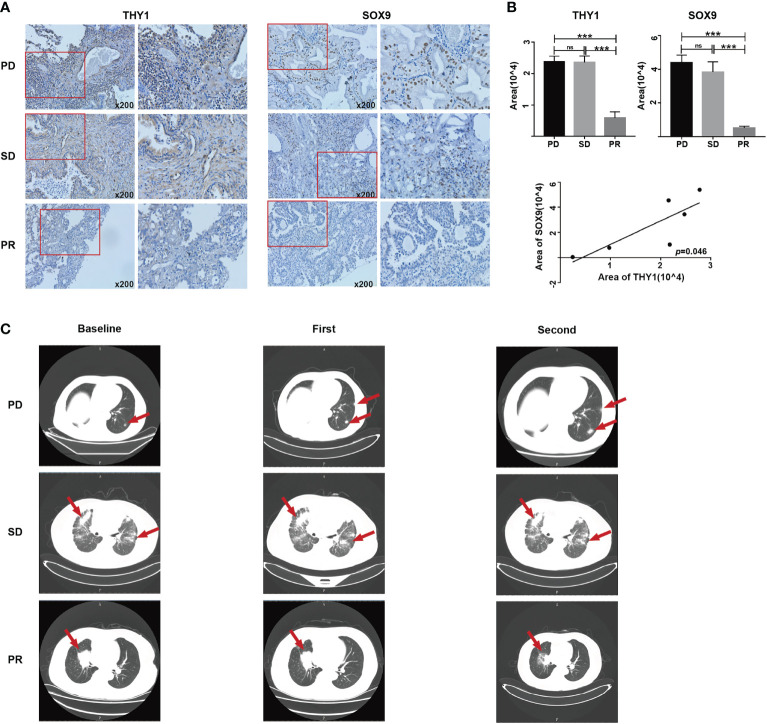
The expression levels of THY1 are significantly associated with SOX9 and efficiency of PD-1 mAb treated LUAD patients. **(A)** Representative immunohistochemical staining of THY1 and SOX9 on tumor tissues of LUAD patient receiving PD-1 mAb treatment. Original magnification, 200×. **(B)** Statistic analysis of immunohistochemical staining score of THY1 and SOX9. Correlation analysis of the immunohistochemical staining score of THY1 and SOX9. ns, no significance; ***p < 0.001. **(C)** Representative CT results of LUAD patients before and after receiving PD-1 mAb treatment. PD, progressive disease; SD, stable disease; PR, partial response.

In order to validate the hypothesis that THY1 is related to cancer stemness and immunotherapy resistance, we performed IHC staining on six tissue samples of LUAD patients who received at least three cycles of PD-1 mAb treatments using THY1 and SOX9 antibodies. The clinicopathological data are presented in **Table 1**. Lung CT examination was used to evaluate the efficacy of PD-1 mAb therapy, and the result prior to the first dose was used as the baseline. The results of the CT scan showed that two patients displayed PD, two patients displayed stable disease (SD), and the remaining two patients acquired partial response (PR) (**Figure 7C**). IHC results proved that THY1 expression was highest in the PD group and lowest in the PR group. Moreover, THY1 expression was positively related to the level of SOX9, a classical biomarker of cancer stemness (38) (**Figures 7A, B**). However, the IHC scores of THY1 and SOX9 between PR group and SD group showed no statistical difference. These results indicated a strong relationship between THY1 and the therapeutic resistance of PD-1 mAb, which was consistent with the above analysis. Furthermore, THY1 levels also positively correlated with cancer stemness. This means that THY1 could be used as a possible biomarker in predicting the efficacy of PD-1 mAb.

## Discussion

The fact that ICI treatments are ineffective for some malignant tumor patients prompts research for the underlying molecular mechanisms and ways to refine it. As a newly developed computational method, TIDE has been used to model tumor immune evasion by evaluating the interactions between candidate genes and the characteristics of T cells in LUAD and melanoma (15). A higher TIDE score is related to a higher likelihood of immune escape by tumors and lower response rate to ICI treatment. As a result, TIDE score is now becoming a more reasonable and accurate method in assessing the outcome of immunotherapy and prognosis of patients compared with the single evaluation of PD-L1 levels, tumor mutational burden, or IFN-γ signature (39). To figure out which cluster of genes is potentially responsible for ICI resistance in LUAD patients, WGCNA was adopted to build a co-expression module and illuminate the complicated correlation between candidate genes and phenotypes. Highly co-expressed genes formed a module that can be used to assess the depth of the association with selected characteristics. Based on these concepts, we used the TIDE score and WGCNA to identify genes associated with immunotherapy resistance in LUAD patients, and picked the pink modules (274 genes), which was highly associated with the TIDE score. In addition, we introduced a cluster of cancer stemness markers (ES1/2, hESC, iPSC, Nanog, Sox2, Myc, et. al) into WGCNA analysis, and observed that these markers were positively correlated with the pink module (24) (**Figure 2B**). These results were in line with previous reports that CSCs mediate resistance to antitumor therapy, such as chemotherapy and radiotherapy, as well as immunotherapy (40–42).

In the exploration of immunotherapy resistance mechanism, cancer stemness has been a focus for years. Maccalli et al. proposed that one main reason for ICI treatment failure might be the immune-resistant ability of CSCs. Although few in number, CSCs remain in the tumor after treatment, reforming the tumor mass and promoting tumor progression (43). In the research by Miranda et al., they found that CD8^+^ T cells, natural killer (NK) cells, and B cells showed a strongly negative correlation with stemness for most cancer (24). These results proposed an association between stemness and suppressed antitumor immune response, which indicates worse clinical outcomes with malignant tumors. Complicated interactions between CSCs and other stromal cells, such as immune cells and fibroblasts, can lead to malignant biological properties such as EMT and result in immune evasion (44). In our analysis, top 15 GO enrichment items of these genes showed a concentration on the organization and regulation of ECM, including EMT (**Figures 3A, B**).

The relationship between ECM remodeling and cancer stemness has already been reported. Elevated collagen-induced stiff ECM triggers cancer cell stem-like programming and metastatic dissemination *in vivo*. Suppression of collagen deposition could increase expression of lung differentiation markers (45). Liang et al. proposed that CD44, a transmembrane receptor for hyaluronic acid and many other ECM components, was a critical marker and regulator of cancer stemness (46). Furthermore, macrophage-derived glycoprotein non-metastatic B facilitates the expansion of CSCs through CD44/IL-33 axis and promote metastasis in mouse tumor models (17). Moreover, ECM could facilitate immune evasion in CSCs by activating PI3K/AKT and recruiting immunosuppressive cells like tumor associated macrophages and Tregs (47–49). The relationship between tumor ECM, drug resistance, and immune suppression has been reported (50). ECM can damage the activation and proliferation of T cells, thus facilitating CSCs survival and inducing immunotherapy resistance (51). Peng et al. demonstrated that LAIR1-SHP-1 pathway regulated the increased collagen levels and exhausted CD8^+^ T cell subpopulations in lung tumor tissues, and mediated the resistance to PD-1/PD-L1 blockade (50). EMT increases tumor initiation and potential for metastasis, as well as resistance to several treatments (52). EMT-associated gene signatures were collectively described as features of intrinsic anti-PD-1 resistance (53). Some studies linked PD-L1 upregulation with EMT, which is also a feature closely related to CSCs (54, 55). Positive correlations were found between EMT-related genes and inhibitory immune molecules (e.g., PD-L1/2, PD-1, CTLA-4) and Tregs in LUAD patients (56, 57). A transcriptomic meta-analysis of breast cancer patients showed that PD-L1 regulated the expression of OCT4A, Nanog, and BMI1 through AKT signaling (58). Thus, EMT is considered to be a potential mechanism in immune escape.

Pathway enrichment analysis in signaling pathways pointed out that genes in the pink module were en1riched in MAPK, JAK-STAT, PI3K, RAS, Notch, Wnt signaling pathways (**Figure 3E**). These pathways have been reported to be activated in immunotherapy resistance. For example, dysregulation of MAPK pathways has been linked with immune-silencing phenotypes in breast cancer and is associated with treatment resistance (59). Activation of the JAK-STAT signaling pathway inhibits cytotoxic T lymphocytes and counteracts the antitumor effects of anti-PD-1 immunotherapy in pancreatic cancer (60). RAS-related oncogenesis could increase the level of PD-L1, eliminate antigen presentation, and alter the expressions of cytokines, thus leading to immune evasion and immunotherapy resistance (61). The research implies a close relationship between immunotherapy resistance and cancer stemness. Further analysis on the immune infiltration patterns of the pink module also confirms these hypotheses. We found that genes in the pink module were positively related with immune suppressive cells (Tregs, Tr1, Tr2, CAF), molecules (CD274), and two critical characteristics in immune evasion (Exclusion and Dysfunction) (**Figure 4B**). Meanwhile, the immune-activated cells and markers, such as antitumor T cells, neutrophils, Th1 cells, and gamma-delta T cells, were downregulated in the pink module (**Figure 4C**). These results support the hypothesis that the genes in the pink module can be used as potential markers or targets in identifying and eliminating malignant tumors.

To ascertain specific genes involved in ICI resistance, we selected nine genes (*COL1A1*, *COL1A2*, *COL3A1*, *COL5A1*, *COL5A2*, *COL6A3*, *EMILIN1*, *MMP2*, *THY1*) for further analysis. These genes are stemness-related and were predicted to be associated with the clinical outcomes of LUAD patients receiving immunotherapy. Expression analysis showed that these genes were more highly expressed in LUAD tumor tissues compared with normal tissues (**Figure 5A**) except for *EMILIN1* and *MMP2*. Moreover, *COL1A1*, *COL1A2*, *COL3A1*, *COL5A1*, *COL5A2*, *THY1* exhibited a more promising outcome in more lowly expressed group (**Figure 5B**). Among them, *THY1* ranked first based on GS.TIDE. THY1 (CD90) is a glycosylphosphatidylinositol-anchored glycoprotein and is mainly expressed on blood stem cells, activated microvascular endothelial cells (ECs), and fibroblasts (62). It could be used as a biomarker in stem cell isolation. THY1 is an important protein in malignant tumors and is considered a candidate marker of CSCs with highly tumorigenic and metastatic potential due to its regulation of cell-cell interactions, cell-matrix interactions, cellular adhesion, and migration (63, 64). Transcriptional analysis of mRNA profiles in CD90^+^ esophageal CSCs showed that CD90-mediated metastasis occurs at least partially *via* the dysregulation of EMT and matrix metalloproteins (64). Strong correlations between THY1 and stemness markers were also found in LUAD according to our research (**Figure 6C**). Except for tumor cells, THY1 has also been observed in stromal cells, like ECs and CAFs, but not CD45^+^ cells (65, 66). Co-localization of THY1 and CD31 implies a possibility that CSCs may reside in the endothelial niche, facilitate vascular formation, and receive supportive signals *via* direct contacts with ECs (67–69). THY1 can influence tumor-infiltrating immune cells. For example, physical interactions between tumor-associated macrophages and CSCs are associated with EMT and could be regulated by CD90 and EphA4 (48). Similarly, THY1 was observed to be positively correlated with immunosuppressive markers, especially TIDE score and T cell exclusion and dysfunction (**Figure 6D**). However, research on THY1 in LUAD is limited. High expressions of THY1 and CD44 influence the relapse-free survival in LUAD patients (70). In our analysis, THY1 was highly expressed in the wound-healing subtype and TGF-β-dominant subtype in LUAD patient (**Figure 6A**). These two subtypes are related to the immunosuppressive phenotype and poor prognosis. Furthermore, THY1 was associated with tumor metastasis, tumor category, and tumor stage in LUAD, which implies that THY1 has an immunosuppressive role in TME and might be responsible for immunotherapy resistance (**Figure 6C**). To verify this hypothesis, we detected the expression of THY1 in LUAD patients who received PD-1 mAb. THY1 was highly correlated with the effectiveness of PD-1 mAb (**Figure 7**). Patients in PD group had the highest expression of THY1 and SOX9, a marker of cancer stemness. These results are consistent with above analysis and strongly support the hypothesis that THY1 might be a predictive marker of immunotherapy resistance and inhibiting it may improve the effects of ICI.

However, there are still some limitations in the current research. Analysis based on a public dataset cannot confirm this result, and the number of LUAD patients receiving PD-1 mAb included in the analysis is small. Moreover, underlying mechanisms of this phenomenon need further research. Therefore, large sample size verification and well-designed biological studies are needed to further confirm our findings.

## Conclusion

In summary, we identified nine hub genes (*COL1A1*, *COL1A2*, *COL3A1*, *COL5A1*, *COL5A2*, *COL6A3*, *EMILIN1*, *MMP2*, *THY1*) as potential participants in implying cancer stemness and immunotherapy resistance. Among them, THY1, which was verified in clinical samples, was associated with the resistance of ICI treatment and co-expressed with familiar CSCs markers. As a result, THY1 may be used as a prognostic LUAD marker; and therapy targeting THY1 may be a promising avenue in eliminating LUAD and enhancing immunotherapy.

## Data Availability Statement

Publicly available datasets were analyzed in this study. This data can be found here: https://www.cancer.gov/tcga and https://www.ncbi.nlm.nih.gov/geo/.

## Ethics Statement

The studies involving human participants were reviewed and approved by the Ethics Committee of the First Affiliated Hospital of Zhengzhou University. The patients/participants provided their written informed consent to participate in this study. Written informed consent was obtained from the individual(s) for the publication of any potentially identifiable images or data included in this article.

## Author Contributions

YZ and LY designed the study and drafted the manuscript. WY and FL prepared the tables and figures and drafted the manuscript. All authors participated in revising the manuscript. All authors contributed to the article and approved the submitted version.

## Funding

This work was supported by grants from the National Natural Science Foundation of China (Nos. 91942314, U1804281, 82072578), the State’s Key Project of Research and Development Plan (No. 2021YFE0110600), the major public welfare projects in Henan Province (201300310400), and the outstanding young talents in science and technology innovation in Henan Province (YXK2020027).

## Conflict of Interest

The authors declare that the research was conducted in the absence of any commercial or financial relationships that could be construed as a potential conflict of interest.

## Publisher’s Note

All claims expressed in this article are solely those of the authors and do not necessarily represent those of their affiliated organizations, or those of the publisher, the editors and the reviewers. Any product that may be evaluated in this article, or claim that may be made by its manufacturer, is not guaranteed or endorsed by the publisher.
